# *SymbioGBR*: a web-based database of *Symbiodinium* associated with cnidarian hosts on the Great Barrier Reef

**DOI:** 10.1186/1472-6785-13-7

**Published:** 2013-03-13

**Authors:** Linda Tonk, Pim Bongaerts, Eugenia M Sampayo, Ove Hoegh-Guldberg

**Affiliations:** 1 School of Biological Sciences, The University of Queensland, St Lucia, QLD 4072, Australia; 2 ARC Centre of Excellence for Coral Reef Studies, The University of Queensland, St Lucia, QLD 4072, Australia; 3 Global Change Institute, The University of Queensland, St Lucia, QLD, 4072, Australia

**Keywords:** *Symbiodinium*, Great Barrier Reef, Coral host, Symbioses, Biogeography

## Abstract

**Background:**

The algal endosymbionts (genus *Symbiodinium*) associated with scleractinian corals (and other reef invertebrates) have received a lot of research attention in the past decade, particularly as certain host-symbiont associations appear more affected by increasing seawater temperatures than others. With the rapid accumulation of information on the diversity of *Symbiodinium*, it is becoming increasingly difficult to compare newly acquired *Symbiodinium* data with existing data to detect patterns of host-symbiont specificity on broader spatial scales. The lack of a general consensus on the classification of *Symbiodinium* species coupled with the variety of different markers used to identify the genus *Symbiodinium* (ITS1, ITS2, LSU D1/D2, chloroplast 23S rDNA and *psbA* minicircle) further complicate direct comparison.

**Description:**

The *SymbioGBR* database compiles all currently available *Symbiodinium* sequences and associated host information of data collected from the Great Barrier Reef into a single relational database that is accessible via a user-friendly, searchable web-based application (http://www.SymbioGBR.org). *SymbioGBR* allows users to query *Symbiodinium* types or sequences sourced from various genetic markers (e.g. ITS1, ITS2, LSU D1/D2 and chloroplast 23S) and invertebrate host species to explore their reported associations. In addition, as the database includes sequence information of multiple genetic markers, it allows cross-referencing between conventional (e.g. ITS2 region) and novel markers that exhibit low intragenomic variability (e.g. *psbA* region). Finally, the database is based on the collection details of individual specimens. Such host-symbiont associations can be assessed quantitatively and viewed in relation to their environmental and geographic context.

**Conclusions:**

The *SymbioGBR* database provides a comprehensive overview of *Symbiodinium* diversity and host-associations on the Great Barrier Reef. It provides a quick, user-friendly means to compare newly acquired data on *Symbiodinium* (e.g. raw sequences or characterized *Symbiodinium* types) with previous data on the diversity of invertebrate host-symbiont associations on the GBR. The inclusion of *psbA*^*ncr*^ sequence information allows for validation of widely used ITS1/ITS2 markers and their ability to accurately identify relevant sequences. Most importantly, centralization of sequence information from multiple genetic markers will aid the classification of *Symbiodinium* species diversity and allow researchers to easily compare patterns of host-*Symbiodinium* associations.

## Background

Symbiotic unicellular dinoflagellates of the genus *Symbiodinium* are best known for their association with scleractinian corals. The symbionts are essential to the functioning of the holobiont by providing their hosts with an important part of their energetic demands [1]. A wide range of reef-dwelling organisms, including the orders Scleractinia, Alcyonacea, Actinaria, Hydroida, Milleporina, Stolonifera, Veneroida, Zoanthidae, Corallimorpharia and Foraminiferida [2-4], depend on their endosymbionts for sufficient energy uptake in oligotrophic tropical seas.

The genus *Symbiodinium* consists of nine broad genetic clades, A-I [5], that were first revealed with small subunit (SSU) rDNA markers [6]. The more variable internal transcribed spacer unit (ITS) 1 and 2 revealed various genetically and ecologically distinct *Symbiodinium* types within these clades: e.g. C1, C3, C21 etc. [3,7-13]. Because of the multi-copy nature and the high intragenomic variance of the ribosomal DNA region, *Symbiodinium* types can contain co-dominant repeats in their genome that manifest as additional bands in the fingerprint pattern and are referred to as intragenomic variants: e.g. C1a, C1b, C1c etc. [2]. While inter-clade differences are substantial and comparable to order-level differences in non-symbiotic dinoflagellate groups [14], genetic distances within *Symbiodinium* clades are generally small. Despite this, closely related *Symbiodinium* types often relate to distinct ecological diversification and influence functional characteristics such as photosynthetic efficiency, growth or thermal tolerance of the host [7,15,16].

The importance of *Symbiodinium* for the holobionts’ (host plus symbionts) stress response is illustrated by the difference in vulnerability to increasing sea surface temperatures (SST) of same host, different symbiont combinations [15,17,18]. While many factors threaten the persistence of coral reefs, increasing SST is regarded as a primary threat to coral reefs by causing disruption of the symbiosis (coral bleaching) and leading to substantial mortality of reef invertebrates over the last two decades [19,20]. The identification of physiological differences in thermal tolerance and bleaching susceptibility between *Symbiodinium* types at the ‘type’ level [17,18] combined with rising SST’s underline the importance of understanding *Symbiodinium* diversity and this has spurred a broad research interest over the past decade [15,18,21-25].

In a recent meta-analysis of *Symbiodinium* data compiled from literature (Tonk et al. unpub. data), 62 different *Symbiodinium* types were identified from 207 host species on the Great Barrier Reef (GBR). Due to its many environmentally distinct areas and broad geographic range (spanning approximately 2300 km and including 10% of coral reefs worldwide) the GBR offers a unique opportunity to study patterns of *Symbiodinium* diversity. With continued efforts to define *Symbiodinium* communities an increasing number of novel types are described [26,27]. As information on cnidarian-*Symbiodinium* symbioses is steadily increasing and expanding its documented geographic extent, it becomes more difficult to compare new with existing *Symbiodinium* data. Although *Symbiodinium* sequences are readily available from generic genetic databases, their usefulness is impeded by the lack of a general consensus on the classification of *Symbiodinium* species. Coupled with the variety of different markers used to identify the genus *Symbiodinium* (internal transcribed spacer region (ITS) 1 and 2, large ribosomal subunit region (LSU) D1/D2, chloroplast 23S rDNA and *psbA* minicircle) it becomes more compelling to assimilate this vastly growing knowledge base into a single, searchable resource.

## Description

We set out to compile currently available sequence and host-association data of *Symbiodinium* reported for the Great Barrier Reef (with the exception of experimentally treated and/or bleached host colonies) into a single relational database that is accessible as a web-based application (http://www.SymbioGBR.org). *SymbioGBR* allows users to query *Symbiodinium* types or sequences, and invertebrate host species to explore symbiotic associations. As the database is based on the collection details of individual specimens, such host-symbiont associations can be assessed quantitatively and viewed in relation to their environmental (e.g., depth) and geographic context (e.g., latitude).

Besides the work involved in compiling previous research, assimilating *Symbiodinium* sequence information has the added difficulty that different techniques (e.g. restriction fragment length polymorphism [RFLP], single-stranded conformation polymorphism [SSCP], denaturing gradient gel electrophoresis [DGGE], direct sequencing) and regions (e.g. 18 s rRNA, LSU D1/D2, ITS1/ITS2, chloroplast 23S) are used for identification. This complicates direct comparison unless the various DNA marker regions are used on a single sample [28]. Another problem arises when the different *Symbiodinium* sequences that are obtained using bacterially cloning of the rDNA are interpreted as if representing distinct *Symbiodinium* types [29]. Due to the multi-copy nature of the rDNA region ecologically relevant sequence variety is easily overestimated by such an approach and these results are likely to confound data generated by DGGE, SSCP, RFLP or direct sequencing [28,30] and are therefor not included in the database.

The database includes sequence information sourced from multiple genetic markers (ITS1, ITS2, LSU D1/D2, chloroplast 23S rDNA and the *psbA* minicircle [*psbA*^*ncr*^]) allowing the use of more regions to create stronger *Symbiodinium* phylogenies. The recent use of the non-coding region of the *psbA*^*ncr*^ was assessed to detect *Symbiodinium* diversity at high resolution [30] and has the advantage that, in contrast to the multi-copy markers of the rDNA region, it’s targeted region is relatively low in intragenomic variation, allowing validation of widely used ITS1/2 markers and their ability to accurately identify dominant sequences. Moreover the added resolution in combination with more conserved genetic markers has the potential to provide the resolution necessary to resolve *Symbiodinium* species classifications [30].

The database will be continuously updated building a unique and growing source of *Symbiodinium* sequence information across regions and cnidarian host-symbiont associations.

## Construction and content

### Data and website development

**Figure 1:**
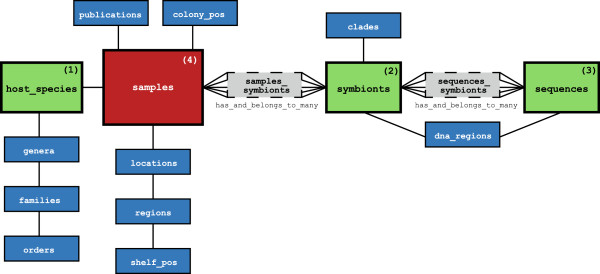
**Database construction and components. **A schematic overview of the relational database model consisting of: four main models (indicated 1 to 4), additional models (in blue) and relationship models linking the main models (in grey).

The *SymbioGBR* data was originally compiled in Microsoft Excel, but was then converted into a relational database model in PostgreSQL. Web interface and model associations were built using Ruby on Rails 3 and a range of Ruby Gems, and the web interface is currently hosted on the cloud platform Heroku. The relational database consists of four main models representing the different host species (1), *Symbiodinium* types (2) and sequences (3), which are connected through an extensive model that contains individual specimen records (4) (Figure 1). The remaining models provide additional standardized information about these data (e.g. source publications, sample location, etc.), in combination with contextual information included in each of the main models (Figure 1).

### *Symbiodinium* sequences and search option

The database supports nomenclature based on both the ITS1 [11,28,31,32] and ITS2 region [2,8,28], but the open format of the database allows for any additional regions to be queried, such as LSU D1/D2, chloroplast 23S rDNA or the *psbA* minicircle. The website contains a dynamically generated “cross-referencing” table (generated with data from samples that were typed using multiple markers), that can aid in comparing *Symbiodinium* diversity across different markers. *Symbiodinium* types that exhibit intragenomic variation that is detectable as additional bands (i.e. contain multiple ITS sequences) [28-30], can be queried through any of these sequences. When sequences are shared between multiple types (e.g., both C3a and C3b share the C3 sequence in their ribosomal genome), the user is offered the various additional sequence options to determine the exact type.

### Source data used in the current version

At the time of publication, the *SymbioGBR* database contains a total of 65 *Symbiodinium* types (sensu LaJeunesse) and at least 219 invertebrate host species that are connected through 4,213 specimen samples. These data are based on 30 source publications, published in the period from 2001 to 2012 [2,4,8-11,17,18,28,30-50].

## Utility and discussion

With the rapid accumulation of information on the diversity of *Symbiodinium* and the growing number of genetic tools, it is becoming increasingly difficult to compare newly acquired *Symbiodinium* data with existing data to detect patterns of host-symbiont specificity on broader spatial scales. This is further complicated by the fact that traditional sequence databases do not accommodate for a classification system with multiple sequences (i.e. representing intragenomic variants [28-30]) per “type” or include cloned sequence data [51]. The need for a web-based database that includes a comprehensive overview of different genetic markers is further illustrated by an increasing awareness that the resolution necessary to support *Symbiodinium* species classification indeed requires such a combination of genetic markers [28,30].

### Query interface

**Figure 2:**
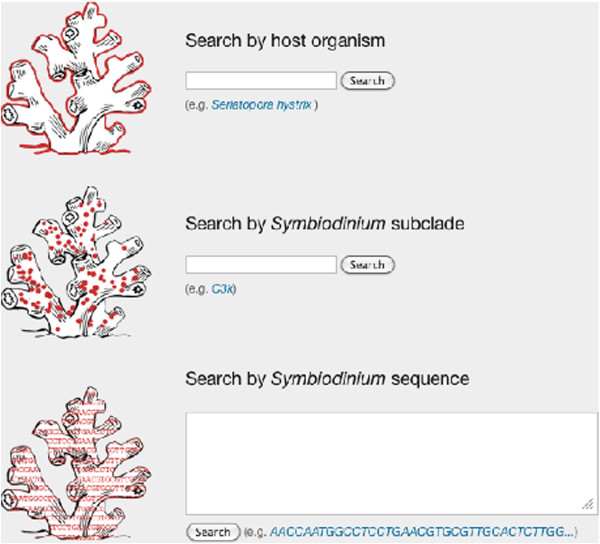
**Query overview. **The home page shows three different search options: host organisms, *Symbiodinium *type, and *Symbiodinium *sequence.

**Figure 3:**
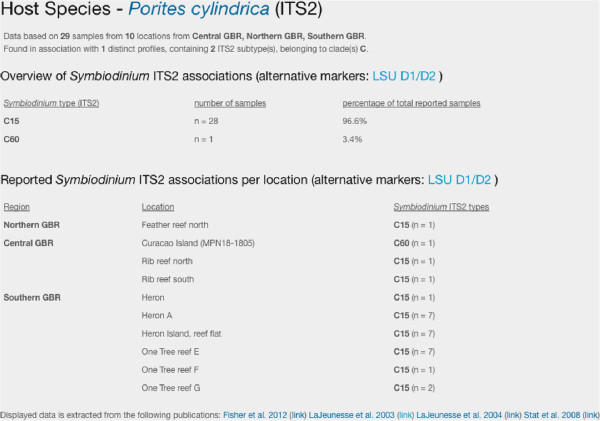
**Host species query. **After submitting *Porites cylindrica *as the search term, hit data are provided in three sections. First a summary is provided followed by an overall overview of the different *Symbiodinium *types harbored by *P. cylindrica *showing the number of host colonies sampled and the percentage of host colonies harboring that particular symbiont type. Finally the different locations are listed alphabetically per section of the GBR showing the *Symbiodinium *types and the number of host colonies sampled. The default setting shows ITS2 data but selecting the desired region in the top right corner can access different DNA regions.

**Figure 4:**
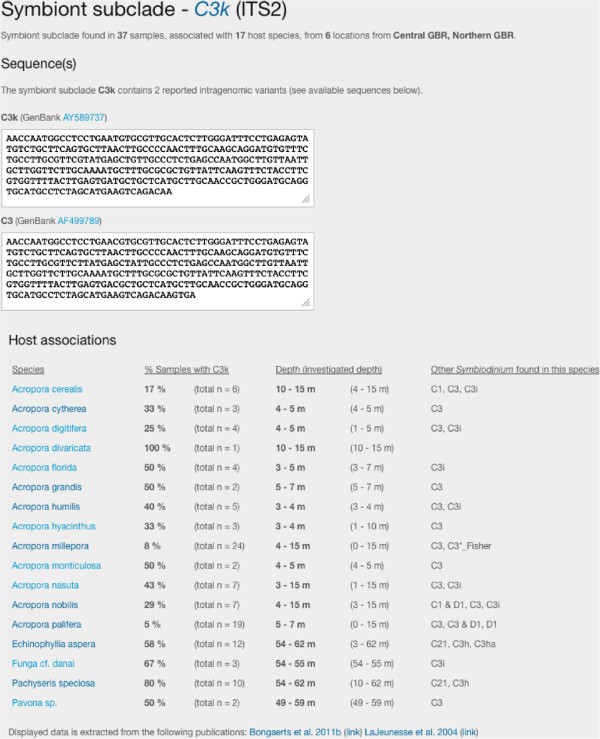
***Symbiodinium *type query. **After submitting C3k as the search term hit a summary is provided followed by sequence information of C3 and C3k in fasta format. Additionally hit data include a table listing the different host species C3k was found in, the percentage and number of colonies with C3k, the investigated depth and other *Symbiodinium* types found in the host.

**Figure 5:**
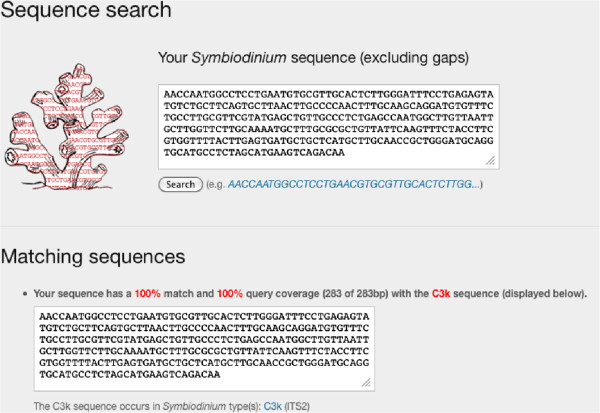
***Symbiodinium *sequence query. **When submitting the C3k sequence in fasta format (excluding gaps) both the submitted *Symbiodinium *type C3k and the matching sequence are displayed as hit data as well as the percentage of matching base pairs and query coverage.

*SymbioGBR* allows users to query *Symbiodinium* types (ITS1/ITS2) or sequences, and invertebrate host species to explore symbiotic associations on the GBR (Figure 2). The option to query host, symbiont type as well as *Symbiodinium* sequence is one of the main features that distinguish *SymbioGBR* from similar type websites [51]. When a host query is performed results are displayed in three sections respectively providing: 1) a summary of the results for that particular host species, 2) a list of the different symbiont types found in the queried host species, and 3) the locations the host species was sampled at per GBR section (Figure 3). *Porites cylindrica* is shown here as an example. The data output shows two *Symbiodinium* types (ITS2) found at ten different locations (Figure 3). When a *Symbiodinium* type query is performed, it will show the different markers that information is available for. After selecting the marker of interest the results are displayed in three sections respectively providing: 1) a summary of the results for that particular symbiont type, 2) sequence information in fasta format with appropriate GenBank accession number, and 3) a list of the different host species in which the symbiont was found (Figure 4). Selecting from the listed host species shows the host species information as described above (Figure 3). For example, C3k data output shows this type is found in 17 host species, mostly from the genus *Acropora* (Figure 4). When a *Symbiodinium* sequence query is performed the matching sequence of the *Symbiodinium* type that particular sequence occurs in is displayed (Figure 5).

### Symbiont identification

Different techniques were used to obtain *Symbiodinium* identity in the various studies that contributed to this database. While DGGE in combination with ITS1/ITS2 was the most commonly used method, other techniques such as RFLP and SSCP were used as well as a suite of different DNA regions (18S rRNA, D1/D2 LSU rDNA, chloroplast 23S rDNA and the *psbA* minicircle) generating variation in the resolution of differentiation, e.g. using the 18S rRNA as a marker allows for comparison at *Symbiodinium* ‘clade’ level whereas ITS markers provide higher resolution and allow for comparative analyses at the *Symbiodinium* ‘type’ level [28]. The website provides an overview of the available sequence information of these various markers used for *Symbiodinium* identification (see Additional file 1) as well as a cross-referencing functionality. The dataset is dominated by ‘type-level’ *Symbiodinium* information using the ITS2 nomenclature *sensu* LaJeunesse, and ITS1 types *sensu* van Oppen. The nomenclature of ITS1 and ITS2 may differ (for example ITS1 type C1 equals ITS2 type C1, ITS1 type C2 refers to ITS2 type C3) and a cross-reference table is provided (see Table 1).

**Table 1:** ITS1/ITS2 translation table

**ITS1 *type***	***Genbank #***	**ITS2 *type***	***Genbank #***	**Comments**
C1_sensu van Oppen	FJ529563	C1	AF333515	
*C1:1a*		*C1*	*AF333515*	*equals ITS1 C1a and ITS2 C1*
*C1:2*	*AF380552*	*C1*	*AF333515*	*equals ITS1 C2 and ITS2 C1*
*C1:3a*	*AY758440*	*C1*	*AF333515*	*equals ITS1 C3a and ITS2 C1*
*C1n*		*C8a*	*FJ529613*	
*C2*	*AF380552*	*C3*	*AF499789*	*sensu van Oppen*
*C3*	*AF380533*	*C3*	*AF499789*	*sensu van Oppen*
*C3a*	*AY758440*	*C1*	*AF333515*	
C3t	FJ529569	C3t	FJ529595	
C33	FJ529566	C33	AY258498	
C33a	FJ529564	C33a	EF541147	
C42a	FJ529561	C42a	FJ529656	
C78a	FJ529562	C78a	FJ529605	
C79	FJ529560	C79	FJ529570	
*Cdot*	*AF180127*	*C15*	*AY239369*	
*Cdot*	*AF180127*	*C17*	*AY239369*	
Cn	AY758555			no ITS2 match sensu van Oppen

Example of the cross-referencing functionality of the website, showing an overview of *Symbiodinium *types that have been identified by both ITS1 and ITS2. Genbank accession numbers are provided when available. *Symbiodinium *types that differ in their ITS1 versus ITS2 nomenclature are indicated in italics. Nomenclature is sensu LaJeunesse unless stated otherwise.

### Database content summary

Host colonies sampled across the different sections of the GBR harboured *Symbiodinium* types from five different clades (clade A, B, C, D, and G). Most host colonies (92%) contained clade C *Symbiodinium* (56 types) which were found in both octocorals and hard corals. Less than 2% of the hosts contained clade D types (D1, D1-4 (formerly known as D1a now assigned a provisional species name, *Symbiodinium trenchi*[22]) and D3) which appear predominantly in hard corals. An additional 5% of the host colonies contained a combination of clade C and D *Symbiodinium* types. Clades A, B, and G were rarely found (1.5%). Clade A was found in fire corals (A7 in *Millepora* spp.) as well as several acroporids (*Acropora longicyathus*, *A. millepora* and *A. valida*). Clade B *Symbiodinium* was restricted to octocorals (B1 and B36 in *Nephthea* spp.) and clade G to octocorals and sponges (but found in a *Stylophora pistillata* colony [50]). Most *Symbiodinium* types (75%) were host specific to either one host species or to several species from the same genus (e.g. C120 in *Seriatopora hystrix* or C17 in the genus *Montipora*). Host generalist types such as C1, C3, C3h and C21 (25% of the *Symbiodinium* types) were found in multiple host genera. The increased resolution portrayed by fine-scale genetic markers, such as the *psbA* minicircle, may reveal that certain host generalist *Symbiodinium* ITS-types in fact represent various host specialist sharing a common lineage at ‘type-level’.

### Limitations & future directions

In order to maintain quality standards and avoid double-naming of new sequences *SymbioGBR* will not provide the option to individually upload information to the web-based database. However, our aim is to keep the database updated with every peer-reviewed publication on *Symbiodinium* diversity on the GBR and we hope that other scientists will assist with contributions to guarantee provision of the most comprehensive and current overview of host-*Symbiodinium* associations on the GBR.

The database is currently dominated by ITS sequence information but has the application in place to provide comparative sequence data of multiple markers used for *Symbiodinium* identification (e.g. LSU D1/D2, chloroplast 23S rDNA and *psbA* minicircle). The accumulation of different markers and their centralization in this database will provide the opportunity to cross-reference between DNA regions (including regions that vary in intragenomic variability) and improve consensus in naming. In addition the cross-reference function provides a means to maintain the applicability of previous work while molecular techniques are continuously evolving.

At this moment *SymbioGBR* is constricted to host-symbiont information from localities within the GBR, one of the most densely studied areas worldwide in terms of *Symbiodinium* diversity. The GBR displays clade C *Symbiodinium* dominance with relatively small contributions of clade A, B, D and G *Symbiodinium*. Conversely, clade C diversity in the GBR is much higher than the diversity found in other clades which is linked to adaptive radiation of clade C in the Indo-Pacific [52]. The template for *SymbioGBR* was constructed in such a way making it suitable for upscaling to an Indo-Pacific or worldwide database if desired.

## Conclusions

The *SymbioGBR* database provides a comprehensive overview of *Symbiodinium* diversity and host-associations on the Great Barrier Reef. As such, it provides a quick means to compare newly acquired data on *Symbiodinium* with previous patterns of diversity and invertebrate host-symbiont specificity and places it in an ecological context. The inclusion of *psbA*^*ncr*^ sequence information allows for further validation of widely used ITS1/ITS2 markers and their ability to accurately identify dominant sequences. Moreover, sequence information sourced from multiple genetic markers allows the use of more regions to create stronger *Symbiodinium* phylogenies and this centralization of sequence information will aid *Symbiodinium* species classification.

## Availability and requirements

The *SymbioGBR* is open to the public on the website http://www.SymbioGBR.org. No license is needed nor are there any restrictions for use by non-academics. The website is a web-application and platform independent.

## Authors’ contributions

LT compiled the information for the database, prepared the sequence alignments, helped to create the website front-end and drafted the manuscript. PB designed the data model, built the relational database application and drafted the manuscript. ES participated in the database compilation, the sequence alignment and helped to draft the manuscript. OHG helped to draft the manuscript. All authors have read and approved the final manuscript.

## Supplementary Material

Additional file 1 **Molecular marker overview.xls – Overview of the different symbiont types and the molecular markers used per type.***Symbiodinium *types are named sensu LaJeunesse unless stated otherwise. When *Symbiodinium *type level is unknown it is indicated as undefined. X indicates the particular marker(s) used to identify the associated type. Click here for file
